# Comparison of quantitative FDG-PET and MRI in anti-LGI1 autoimmune encephalitis

**DOI:** 10.1007/s00234-023-03165-2

**Published:** 2023-06-02

**Authors:** Mohammad S. Sadaghiani, Samantha Roman, Luisa A. Diaz-Arias, Ralph Habis, Arun Venkatesan, John C. Probasco, Lilja B. Solnes

**Affiliations:** 1 Division of Nuclear Medicine and Molecular Imaging, The Russell H. Morgan Department of Radiology and Radiological Science, Johns Hopkins University School of Medicine, 601 N. Caroline St., Baltimore, MD 21287 USA; 2 Department of Neurology, Johns Hopkins University School of Medicine, 601 N. Caroline St., Baltimore, MD 21287 USA

**Keywords:** Anti-LGI1, Autoimmune encephalitis, FDG PET/CT

## Abstract

**Objectives:**

Anti-leucine glioma-inactivated protein 1 (anti-LGI1) autoimmune encephalitis (AE) presents as subacute memory loss, behavioral changes, and seizures. Diagnosis and treatment delays can result in long term sequelae, including cognitive impairment. ^18^F-FDG PET/CT may be more sensitive than MRI in patients with AE. Our objective was to determine if anti-LGI1 is associated with a distinct pattern of FDG uptake and whether this pattern persists following treatment.

**Methods:**

Nineteen^18^F-FDG PET/CT brain scans (13 pre-treatment, 6 convalescent phase) for 13 patients with anti-LGI1 were studied using NeuroQ™ and CortexID™. The sensitivity of the PET images was compared to MRI. The Z scores of 47 brain regions between the pre-treatment and next available follow-up images during convalescence were compared.

**Results:**

All ^18^F-FDG PET/CT scans demonstrated abnormal FDG uptake, while only 6 (42.9%) pre-treatment brain MRIs were abnormal. The pre-treatment scans demonstrated hypermetabolism in the bilateral medial temporal cortices, basal ganglia, brain stem, and cerebellum and hypometabolism in bilateral medial and mid frontal, cingulate, and parietotemporal cortices. Overall, the brain uptake during convalescence showed improvement of the Z scores towards 0 or normalization of previous hypometabolic activity in medial frontal cortex, inferior frontal cortex, Broca’s region, parietotemporal cortex, and posterior cingulate cortex and previous hypermetabolic activity in medial temporal cortices, caudate, midbrain, pons and cerebellum.

**Conclusions:**

Brain FDG uptake was more commonly abnormal than MRI in the pre-treatment phase of anti-LGI1, and patterns of dysmetabolism differed in the pre-treatment and convalescent phases. These findings may expedite the diagnosis, treatment, and monitoring of anti-LGI1 patients.

## Introduction

Anti-leucine-rich glioma inactivated 1 (anti-LGI1) autoimmune encephalitis (AE) is a subset of antibody-related encephalitis manifesting clinically as subacute onset memory impairment, behavioral changes, and seizures including facial brachial dystonic seizures [1, 2]. LGI1 is a secreted neuronal protein that forms a trans-synaptic complex involving two disintegrin and metalloproteinase domain-containing proteins (presynaptic ADAM23 and postsynaptic ADAM22), presynaptic voltage-gated potassium channels Kv1.1, and postsynaptic AMPA receptors [3]. The clinical presentation is most commonly reflective of limbic encephalitis. Up to two thirds of patients will demonstrate increased signal in the medial temporal lobes on MRI [4]. It has been shown that during the acute phase MRI is abnormal in 74% of the patients with Anti-LGI1 AE [2].

Early recognition of AE is imperative as early intervention is thought to lead to better clinical outcomes for patients [5]. This is particularly well-established for anti-LGI1 AE [6]. The diagnosis is complex as there are many etiologies and clinical presentations that can mimic AE [7]. Classically, diagnosis involved serum and cerebrospinal fluid antibody testing which can take weeks, potentially delaying initiation of therapy and leading to worse outcomes. In 2016, expert consensus clinical criteria were developed to facilitate early recognition of AE and expedite the initiation of immunotherapy [8]. These clinical consensus criteria included designations of possible, probable, or definite AE as well as definite limbic encephalitis based on clinical presentation and electroencephalographic, cerebrospinal fluid, and brain MRI findings [8]. The criteria for definite limbic encephalitis also included medial temporal lobe hypermetabolism on brain ^18^F-FDG PET [8]. Subsequent best practice recommendations have included brain and body ^18^F-FDG PET in the diagnosis and acute management of AE in general [5].

Recent literature has supported the utility of ^18^F-FDG PET brain studies in aiding with the diagnosis of AE, including several specific AE syndromes [9-11]. Utilizing CortexID™ for semiquantitative analysis, we previously found that ^18^F-FDG PET CT was abnormal in 85% of patients with AE, most commonly demonstrating brain region hypometabolism [9]. Additional analysis of this group showed that ^18^F-FDG PET CT is more likely to identify an abnormality than MRI of the brain [10]. Further studies are required for different subsets of AE to determine whether a definitive pattern of hypo- or hypermetabolism in the brain is associated with specific antibodies. Early findings suggest that certain antibody-related AEs present with specific patterns of abnormal brain metabolism on PET. We have reported that patients with anti-NMDA receptor encephalitis present with occipital lobe hypometabolism early in the disease course, coinciding with greater neurologic disability [11]. A recent study evaluated 33 patients with anti-LGI1 AE who underwent ^18^F-FDG PET imaging of the brain, demonstrating a myriad of metabolic findings not confined to the temporal lobes and basal ganglia [12].

In this study, we utilize commercially available software to perform semiquantitative analyses of cortical and subcortical regions of interest to describe the ^18^F-FDG PET findings in patients with anti-LGI1 AE prior to treatment, and compare the sensitivity of FDG-PET to brain MRI. We also characterize and compare the cerebral metabolism patterns prior to treatment with those during convalescence.

## Materials and methods

This study was performed according to STARD 2015 guideline and was approved by the Institutional Review Board of Johns Hopkins University.

### Patients

We retrospectively identified patients admitted to the Johns Hopkins Hospital with confirmed AE who underwent ^18^F-FDG PET/CT using the diagnostic terms “encephalitis” and “positron emission tomography” (PET) to search the administrative database (December 1, 2005–March 15, 2016) and cross-referenced these patients with the Johns Hopkins Hospital PET/CT Center database [9]. We also included patients prospectively enrolled in an encephalitis patient registry through November 1, 2022.

Included patients were found to have definite AE (including definite limbic encephalitis) per clinical consensus criteria [8] and were seropositive for the anti-LGI1 antibody per serum and/or CSF testing (Athena Diagnostics, Worcester, MA; Mayo Clinic Laboratories, Rochester, MN). Demographic information, neurological symptoms at time of FDG-PET/CT during initial admission and subsequent care, diagnostic test results including autoimmune encephalopathy antibody assays of the serum and CSF, and treatments were reviewed from the electronic medical record.

### ^18^F-FDG PET acquisition and processing

Resting brain ^18^F-FDG PET/CT images were acquired in 3-dimensional (3D) mode for 10 min on a Discovery DRX or DLS (GE Healthcare; 20 patients) or a Biograph mCT (Siemens; 3 patients) with in-line CT for attenuation correction. Filtered back-projection reconstructions of the 10-min brain ^18^F-FDG PET acquisitions were used for quantitative analysis.

Semiquantitative analysis of PET images (including pre-treatment and convalescent images) was performed using CortexID™ (GE Healthcare, Chicago, MI, USA) and NeuroQ™ (Syntermed, Atlanta, GA, USA). In both CortexID™ and NeuroQ™, activity in each voxel was normalized to the pons. Our prior studies have been based on CortexID™; however, we have observed more relevant results with NeuroQ™ which is a more recent platform and to have a grasp of their output we included both in our analysis. NeuroQ™ provides regional assessment of human brain scans based on automated quantification of mean pixel values lying within standardized regions of interests (ROIs) and compares the brain regional activity to normal activity values of 50 normal controls. Forty-seven areas in the brain were segmented and analyzed. All pre-treatment PET images were analyzed with NeuroQ™ and CortexID™, except for patient 11 which the number of slices in PET images were not compatible with CortexID™ and for whom only analysis with NeuroQ™ was performed.

CortexID™ provides the Z scores of 29 different brain regions. CortexID™ calculates automated voxel-by-voxel Z scores using the following formula: (Z score = [mean database − mean subject]/SD database) however this formula in NeuroQ™ is implemented as follows (Z score = [mean subject − mean database]/SD database). As a result of the difference in the numerator of the formula between NeuroQ™ and CortexID™, the Z scores below 0 are considered hypometabolic and above 0 are hypermetabolic in NeuroQ™; however, in CortexID™, regions with values above 0 are hypometabolic and regions with values below 0 are hypermetabolic. To visually represent the relative metabolism of different brain regions, heat maps were generated. Shades of green are used for hypermetabolic, and shades of red are provided for hypometabolic region in both CortexID™, and NeuroQ™. We considered Z score 1.65 as a threshold of abnormality, as per instruction provided by NeuroQ™. The author (M.S.) who did the semiquantitative analysis was aware of the diagnosis of the patients.

### Brain MRI

Both the images and the radiology reports for contrasted brain MRI completed around the time of the acute phase ^18^F-FDG PET scan were reviewed by a board-certified radiologist with neuroradiology training (L.S.).

### Statistical analysis

**Fig. 1:**
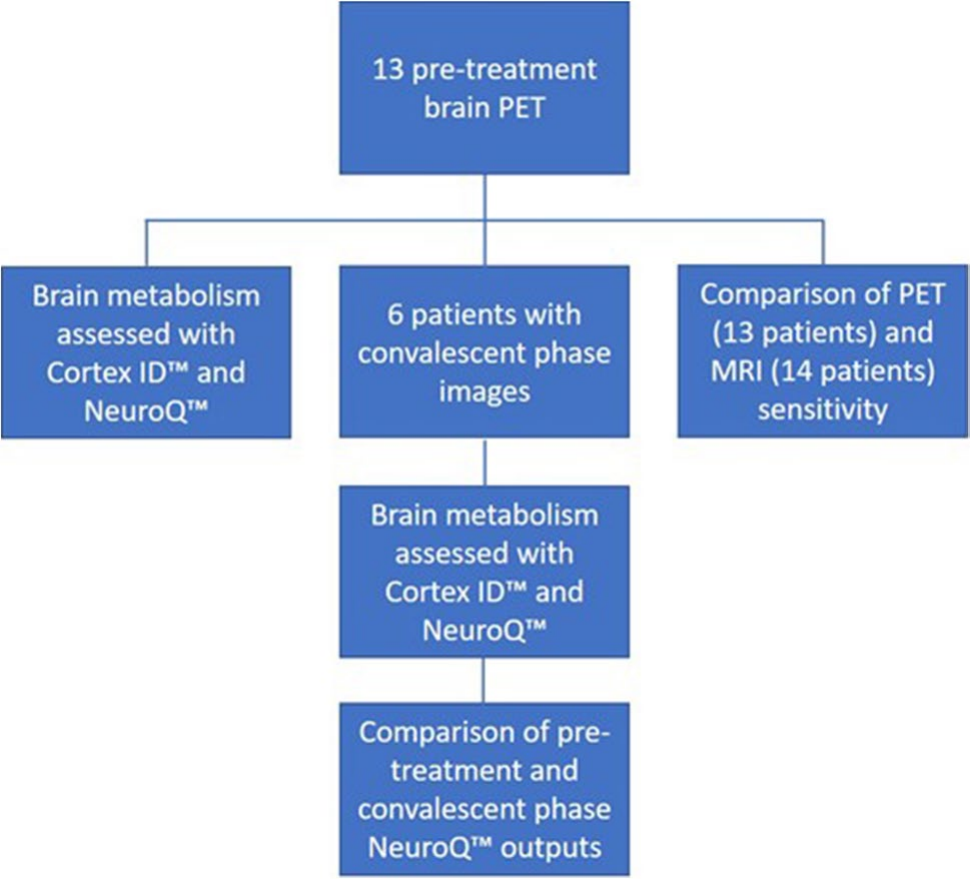
Diagram showing the steps of the study

Using the NeuroQ™ output, we compared the Z scores of 47 brain regions between the acute and next available follow-up images during the convalescent phase. In addition, based on literature review and pathophysiology of faciobrachial dystonic seizure we compared the Z scores of the cerebellum and basal ganglia between patients with faciobrachial dystonic seizures and those without this symptom. The statistical analyses were performed with Stata version 17.0 (StataCorp, College Station, Texas), and *p* values less than 0.05 were considered significant. The sensitivity of PET and MR images were evaluated in detecting abnormal findings among pre-treatment patients (Fig. 1).

## Results

### Patients

Fourteen patients with Anti-LGI1 AE were identified and included (Table 1). The mean age of the patients was 68.4 ± 18.6. Eight (57%) of the patients were male and 10 (71%) were of white race. Thirteen of the patients underwent initial brain ^18^F-FDG PET/CT scans within a median of 10 weeks after symptom onset (range 2–24 weeks). Six patients had follow-up scans during convalescence (median 60.5 weeks from original syndrome onset, range 36–91 weeks). A total of 19 scans were evaluated (13 pre-treatment and 6 convalescent). All patients had brain MRI scans during the acute phase of illness.

**Table 1:** Demographics, clinical characteristics, and initial clinical management of patients with anti-LGI1 encephalitis who underwent FDG-PET/CT and/or MRI through the course of their inpatient evaluation. mRS, modified Rankin score for disability; NA, not applicable

Patient	Gender, age (years)	Race	Duration of symptoms prior to FDG-PET/CT (weeks)	Presence of Faciobrachial dystonic seizures on admission	Additional clinical signs and symptoms	Brain MRI features	Initial brain MRI findings	EEG findings	CSF findings (WBC/cc, protein mg/dL)	Initial medical treatment	mRS at admission, discharge (impression relative to admission), and 6 month follow-up (impression relative to admission)
1	Female, 85	White	4	Yes; bilateral face and arms	Impaired short-term memory, delusions, falls, increased appetite and oral intake; hyponatremic on admission	3 T FLAIR: 4 mm slice thickness, TE 105, TR 9000	No acute abnormalities, chronic small vessel ischemic changes	Occasional semirhythmic to rhythmic delta activity diffusely with frontal predominance, no seizures nor epileptiform discharges	1, 31	IVMP, valproate	3, 2 (improved), 1 (improved)
2	Male, 62	White	4	Yes; bilateral face and arms	Impaired short-term memory, abnormal behavior, generalized tonic-clonic seizure, delusional, visual and auditory hallucinations, nonsensical speech, insomnia; hyponatremic on admission	3 T FLAIR: 4 mm slice thickness, TE 105, TR 9000	Normal brain parenchyma with diffuse pachymeningeal enhancement following lumbar puncture	Left and right temporal lobe seizures, independent left and right periodic lateralized epileptiform discharges	2, 49	IVMP, IVIg, phenytoin	3, 2 (improved), 0 (improved)
3	Male, 61	White	8	Yes; right face and arm	Impaired short-term memory, emotional lability, fatigue, aphasia, startle episodes, episodes of hesitation with ambulation; hyponatremic on admission	3 T FLAIR: 4 mm slice thickness, TE 105, TR 9000	Normal study	Bilateral, symmetric posterior basic rhythm slowing	10, 79	PLEX, levetiracetam, lorazepam	2, 2 (stable), 1 (improved)
4	Male, 66	White	12	No	Impaired short-term memory, shiver events, fatigue, report of myoclonus with initial events; hyponatremic on admission	1.5 T FLAIR: 5 mm slice thickness, TE 125.8, TR 8802.0	T2/FLAIR hyperintensity with mild sulcal effacement in bilateral medial temporal lobes			PLEX, lamotrigine, lacosamide	2,2 (stable), 1 (improved)
5	Male, 76	White	14	Yes; left face, arm, and leg	Impaired short-term memory, word finding difficulty, impaired calculations, hallucinations on waking, de ja vu episodes, metallic taste in mouth prior to as well as gait instability and falls after faciobrachial dystonic seizures, hyponatremic on admission	3 T FLAIR: 4 mm slice thickness, TE 105, TR 9000	No acute abnormalities, right insular/subinsular developmental venous anomaly	Normal Study	2, 49	IVMP, PLEX, levetiracetam	3, 3 (stable), 2 (improved)
6	Male, 67	Other	10	No	Impaired short-term memory, poor navigation, dysarthria, cold sensation descending from head to chest, generalized tonic clonic seizure, myocardial infarction; hyponatremic on admission	1.5 T FLAIR: 5 mm slice thickness, TE 90, TR 8000 cortex, without enhancement	Mildly restricted diffusion involving the right caudate extending into the right putamen, with a punctate focus in the right frontal lobe cortex, without enhancement	Normal study	1, 45	IVMP, IVIG, PLEX, levetiracetam, valproate	3, 2 (improved), 2 (improved)
7	Male, 65	Hispanic	8	No	Impaired short-term memory, confusion, difficulty with calculations, increased anxiety, personality changes, hyponatremic on admission	3 T FLAIR: 4 mm slice thickness, TE 105, TR 9000	T2/FLAIR hyperintensity with associated subtle contrast enhancement involving both amygdala and hippocampi	Mild posterior basic rhythm slowing, more prominent on left	4, 35	IVMP, IVIg, PLEX, levetiracetam, risperidone	3, 3 (stable), lost to follow-up after hospital discharge
8	Female, 86	White	12	Yes; bilateral face, arm, and leg	Impaired short term followed by long term memory loss, visual and tactile hallucinations, cessation of speech during faciobrachial dystonic seizures; hyponatremic on admission	1.5 T FLAIR: 4 mm slice thickness, TE 100, TR 9000	T2/FLAIR hyperintensity in right medial frontal lobe and right anterior cingulate gyrus with gyriform enhancement on postcontrast images; T2/FLAIR hyperintensity of the right basal ganglia with contrast enhancement	Diffuse slowing, more prominent over right anterior head regions	3, 34	IVMP	5, 4 (improved), 4 (improved)
9	Female, 75	White	8	Yes; bilateral face and arms	Impaired short-term memory, lightheadedness, tensing in throat prior to and cessation of speech with confusion after faciobrachial dystonic seizure	1.5 T FLAIR: 4 mm slice thickness, TE 101, TR 9000	Normal sudy	Left frontotemporal slowing	3, 113	IVMP, IVIg, levetiracetam	2,2 (stable), 0 (improved)
10	Male, 59	White	24	No	Wide-based and shuffling gait, anxiety, impaired attention and short-term memory, urinary incontinence, hyponatremic on admission	3 T FLAIR: 3 mm slice thickness, TE 94, TR 9000	Mild cerebral volume loss, chronic small vessel ischemic changes in the supratentorial white matter, mild ventriculomegaly	Normal study	1, 101	IVMP, PLEX, lacosamide	4, 3 (improved), 3 (improved)
11	Female, 63	Asian	24	Yes; left arm and leg	Impaired short-term memory, seizures, fatigue, insomnia, anorexia, hyponatremic on admission	3 T FLAIR: 4 mm slice thickness, TE 119, TR 9000	Bilateral T2/FLAIR hyperintensities of the mesial temporal lobes and hippocampus	Left temporal lobe seizures with hyperventilation	1, 36	PLEX, lacosamide	3, 2 (improved), 2 (improved)
12	Male, 55	Other	2	Yes; right arm	Confusion, impaired short-term memory, shock-like sensation in right arm, hyponatremic on admission	3 T FLAIR: 5 mm slice thickness, TE 119, TR 8000	Normal	Left temporal lobe seizures, interictal left temporal lobe slowing	11, 65	IVMP, PLEX, valproate, lacosamide, levetiracetam	3, 3 (stable), 2 (improved)
13	Female, 66	White	13	No	Impaired short-term memory, episodes of staring, fatigue, day-time sleepiness, hallucinations, déjà vu, dizziness, palpitations	3 T FLAIR: 4 mm slice thickness, TE 90, TR 8800	Normal	Bilateral posterior epileptiform activity triggered by eye closure and photic stimulation, right occipital epileptiform discharges	WBC NA, 27	Prednisone, IVMP, PLEX, levetiracetam	3, 2 (improved), 2 (improved)
14	Female, 72	White	NA	Yes; right face and arm	Impaired attention and short-term memory, personality change, episodes of unresponsiveness, waking from sleep with startle, fatigue, excessive sleepiness, hyponatremic on admission	3 T FLAIR: 4 mm slice thickness, TE 95, TR 1200	T2/FLAIR hyperintensity and gyral enlargement of right greater than left mesial temporal lobes	Left temporal epileptiform discharges, slow background, intermitted rhythmic slowing	15, 98	IVMP, levetiracetam	3, 2 (improved), 2 (improved)

In addition to impaired short-term memory and faciobrachial dystonic seizures which are presented in Table 1, the patients presented a variety of additional signs and symptoms including abnormalities of speech, hallucinations, delusions, seizures, abnormalities of gait and/or falls, and hyponatremia. Brain MRI demonstrated abnormalities of the medial temporal lobes in four (patients 4, 7, 11, 14) and the basal ganglia in two patients (6, 8). EEG was abnormal in 10 patients, with abnormalities varying from slowing of the posterior basic rhythm to bilateral temporal lobe seizures. Only three patients were found to have a CSF pleocytosis (3, 12, 14). All patients were treated with first-line immunosuppressive therapy, and 13 were treated with anti-seizure medications.

### Pre-treatment ^18^F-FDG PET semiquantified analysis

**Fig. 2:**
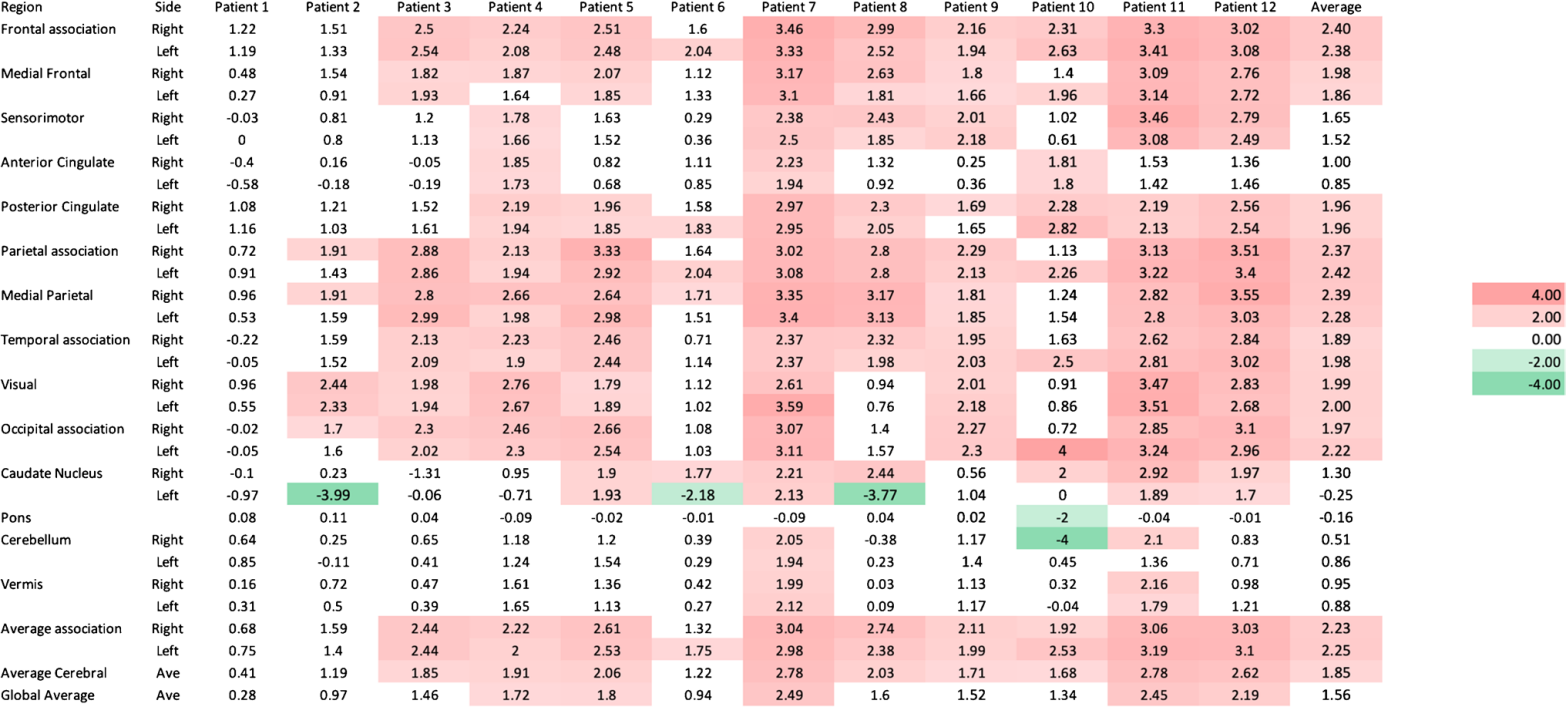
Heat map showing pre-treatment Z scores from CortexID^TM^ color-coded to green (hypermetabolism) or red (hypometabolism)

**Fig. 3:**
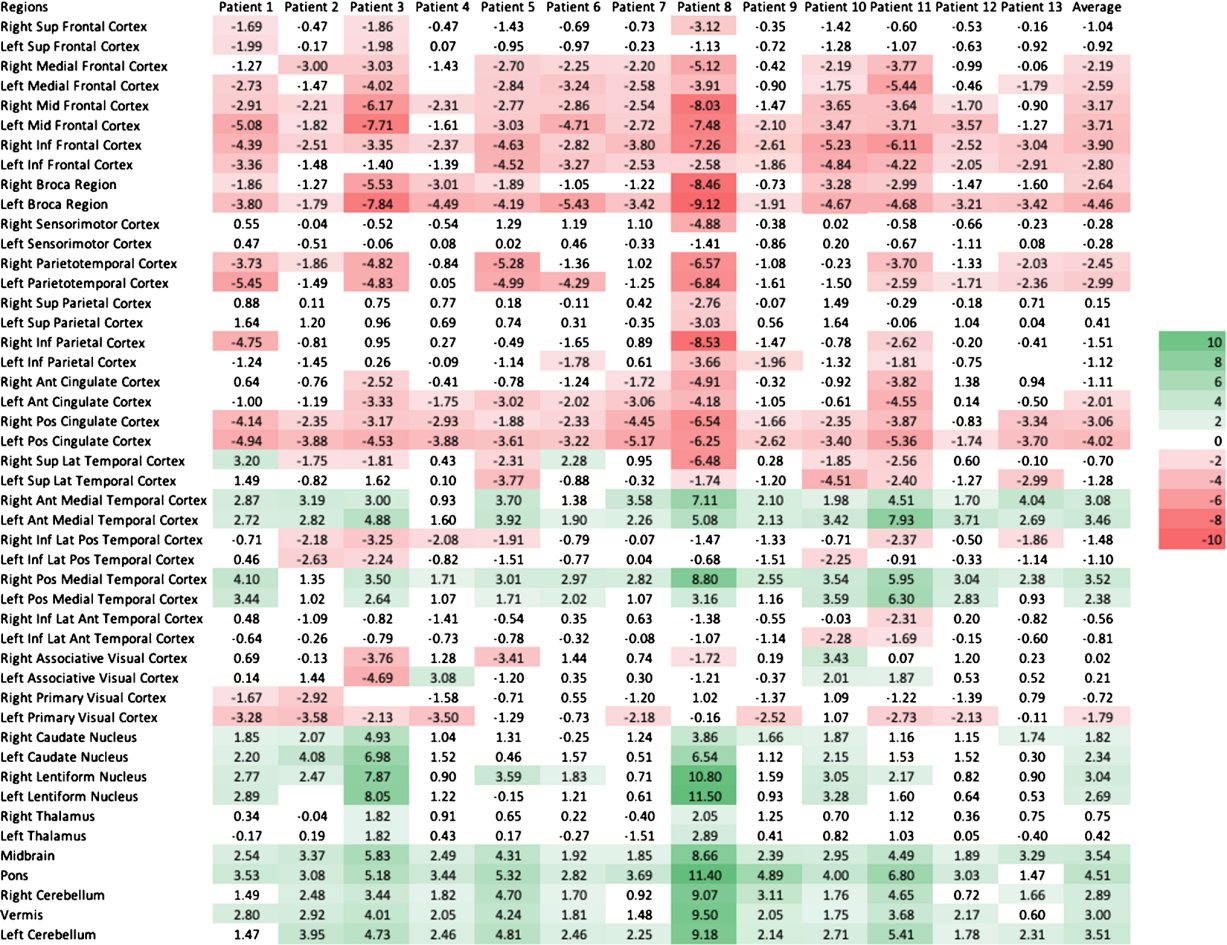
Heat map showing pre-treatment Z scores from NeuroQ™ color-coded to green (hypermetabolism) or red (hypometabolism) for 47 different brain regions

**Fig. 4:**
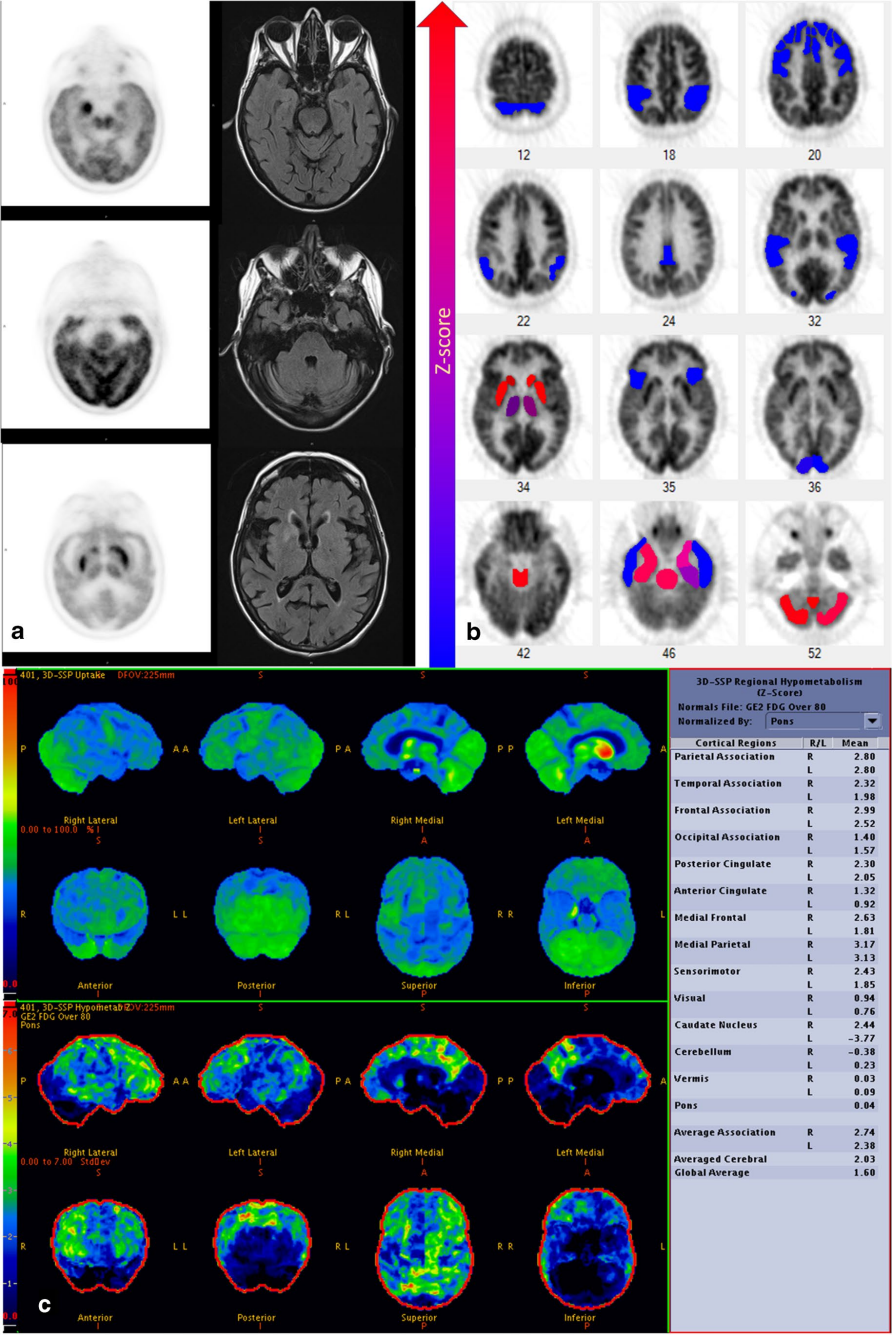
Brain MRI and brain 18F-FDG-PET in a patient with anti-LGI1 autoimmune encephalitis.** a** MRI images show FLAIR hyperintense foci corresponding to areas of high FDG avidity on PET images in the bilateral basal ganglia, and right medial temporal lobe. In addition, there is symmetric high FDG avidity of bilateral cerebellum, and brain stem. There is mild cortical expansion of the right medial temporal lobe. **b** NeuroQ™ output with abnormal hypermetabolic regions including the bilateral basal ganglia (slice 34), medial temporal lobes (slice 46), brain stem (slice 46), and cerebellum (slice 52). **c** CortexID™ output that did not detect the abnormal uptake in the medial temporal lobes and cerebellum. CortexID™ output showed right caudate hypo and left caudate hypermetabolism. The positive Z score values above 1.65 in CortexID^TM^ demonstrate hypometabolism

Evaluation of the pre-treatment images with CortexID™ and NeuroQ™ showed similar patterns in the frontal and parietal lobes. However, comparison of the temporal lobes, and cerebellum showed considerable discrepancy between these two platforms (Figs. 2 and 3). Visual inspection of the PET images showed that NeuroQ™ accurately detects small regions of dysmetabolism while CortexID™ did not detect abnormal uptake. For example, Fig. 4 depicts a patient whose PET images showed abnormal high FDG avidity in the bilateral medial temporal lobes, basal ganglia, brainstem, and cerebellum while CortexID™ only detected hypermetabolism in the left caudate nucleus (patient 8) and hypometabolism in the bilateral temporal lobes. Based on visual inspection of the images we will focus on the results of NeuroQ™ in this study.

NeuroQ™ showed abnormal ^18^F-FDG uptake among all patients in the pre-treatment phase in most brain regions. The pre-treatment scans demonstrated abnormal hypermetabolism in the medial temporal cortices, caudate and lentiform nuclei, midbrain, and cerebellum, while hypometabolism was seen in the bilateral medial and mid-frontal, parietotemporal, cingulate cortices, and bilateral Broca regions. All patients showed at least one area of hypermetabolism among the following regions: right anterior, left anterior, right posterior, and left posterior medial temporal lobes. Figure 3 is a heat map based on NeuroQ™ output that color-codes the Z score values of each of the 47 brain regions from the baseline scans of each patient, as well as the average values among the included patients.

### ^18^F-FDG PET analysis comparing pre-treatment versus convalescent images

**Fig. 5:**
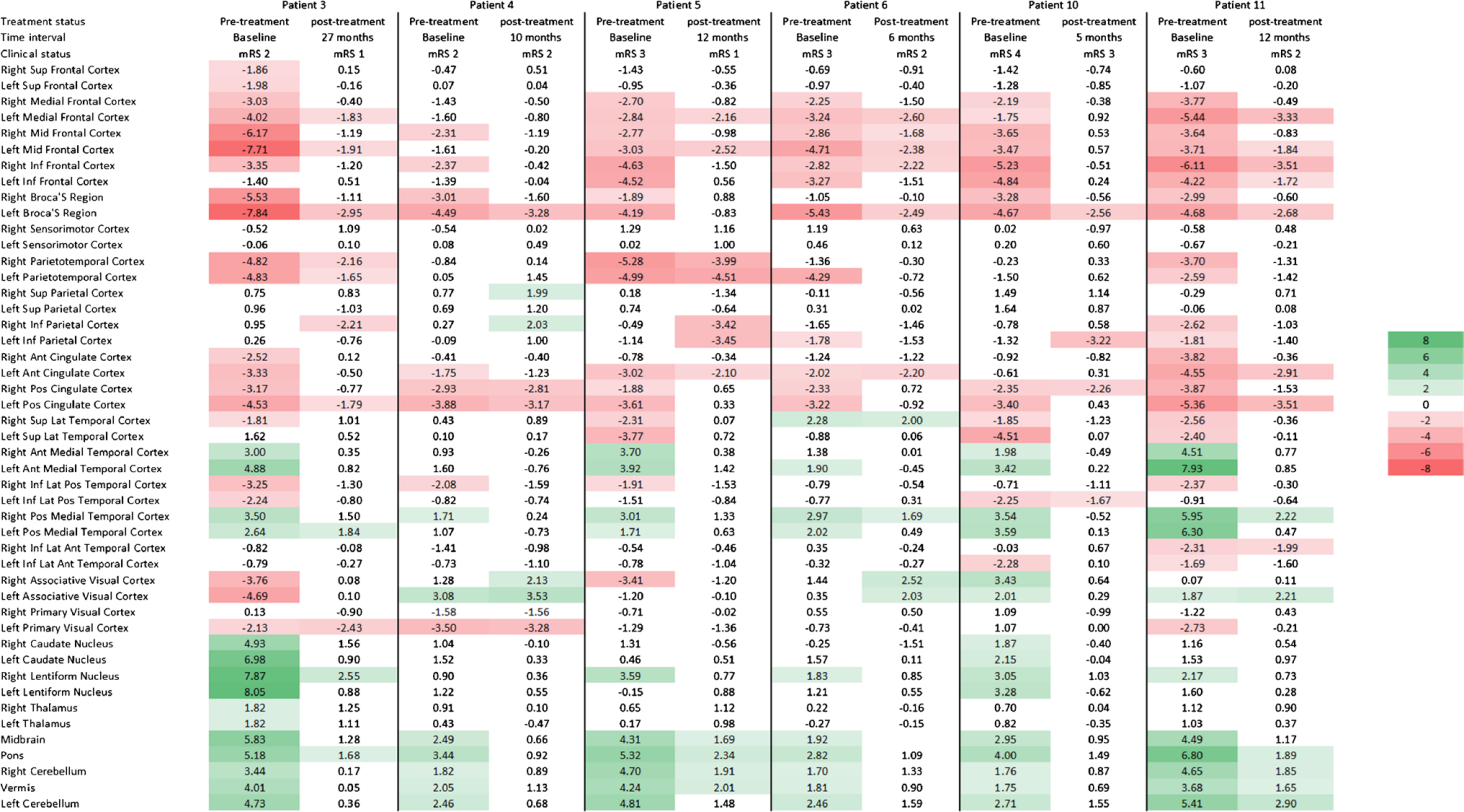
Heat map showing pre-treatment and convalescent phase Z scores from NeuroQ™ color-coded to green (hypermetabolism) or red (hypometabolism) for 47 different brain regions in 6 patients with follow up imaging

**Fig. 6:**
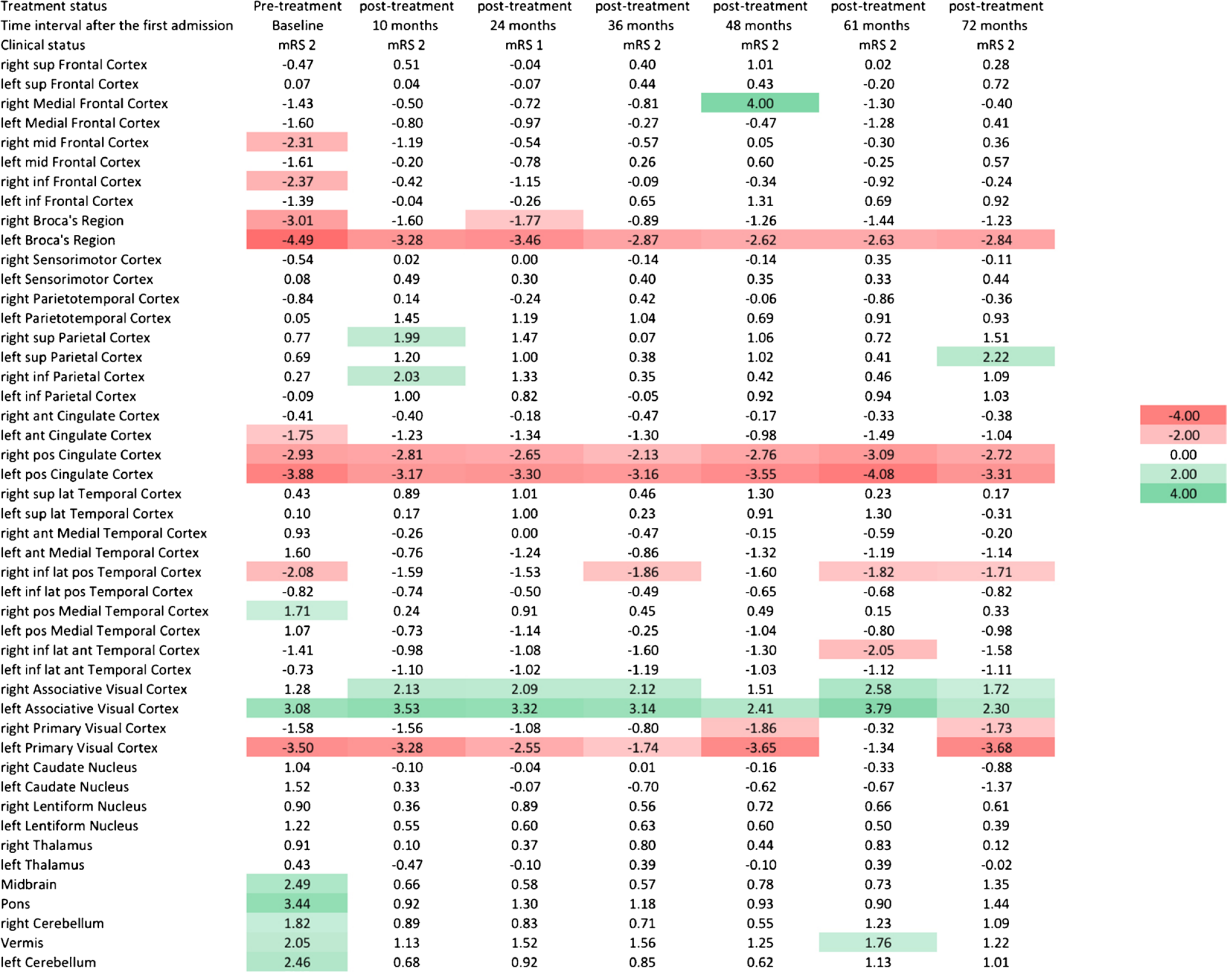
Heat map showing the Z score values from NeuroQ^TM^ of 47 different brain regions among the multiple follow up PET images for patient 4

**Fig. 7:**
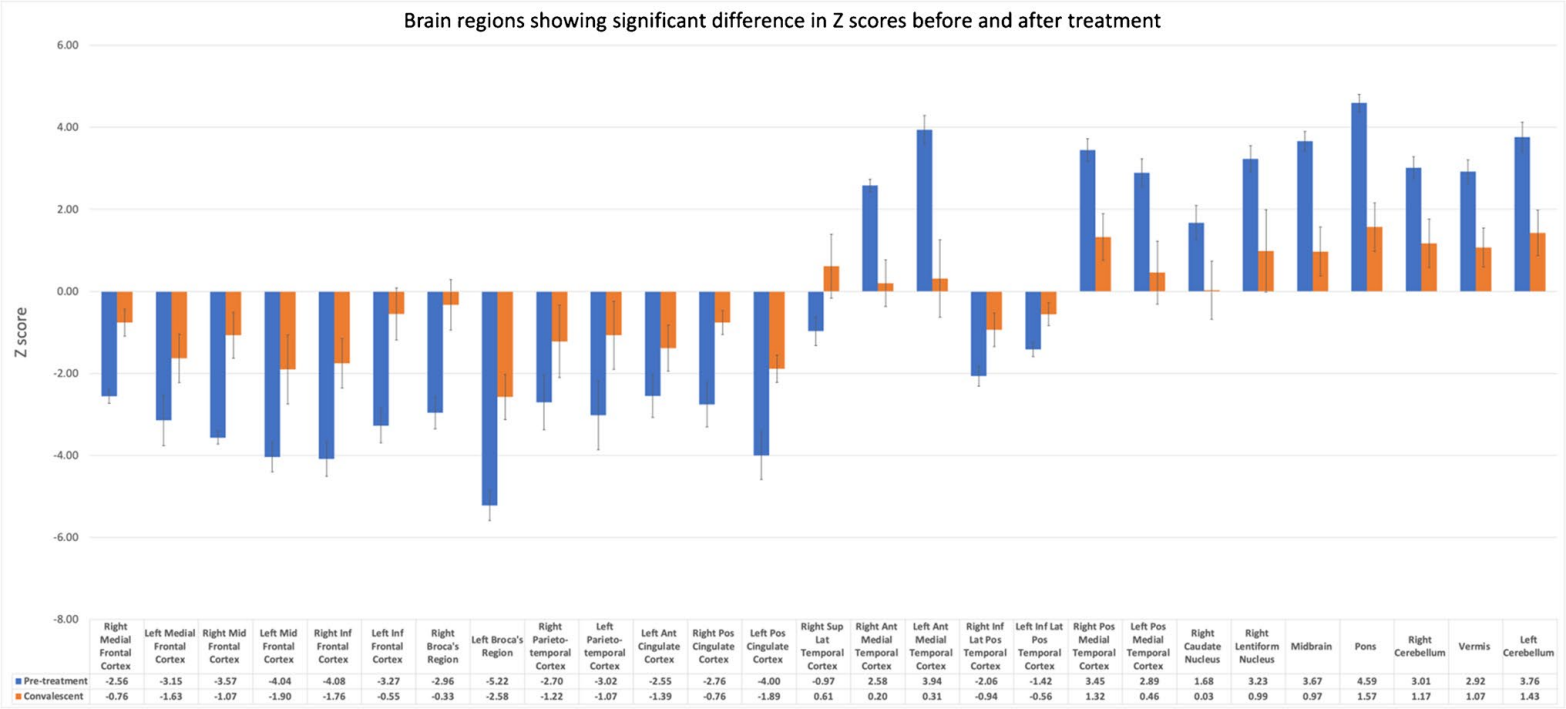
Brain regions with significant difference in Z scores between the pre-treatment and convalescent phases using NeuroQ

Using the NeuroQ™ output, the comparison of the pre-treatment phase images with subsequent convalescent studies (Fig. 5) showed improvement of most of the abnormal regions among all 6 patients with follow-up imaging, including normalization of Z scores in areas previously hypometabolic (such as right medial frontal cortex, right inferior frontal cortex, left Broca’s region, right parietotemporal cortex, left posterior cingulate cortex) and hypermetabolic (such as bilateral medial temporal cortices, right caudate nucleus, midbrain, pons, and left cerebellum) on pre-treatment images (Fig. 5). Among the 6 patients with follow up imaging, 5 of them showed clinical improvement at the time of follow up PET study and only patient #4 remained stable clinically. This patient had 6 follow-up studies and the Z scores of the different time intervals are provided in Fig. 6. Twenty-seven brain regions showed statistically significant differences between the pre-treatment and convalescent phases (using Wilcoxon signed-rank test) which is demonstrated in Fig. 7.

### ^18^F-FDG PET analysis comparing faciobrachial dystonic seizures with patients without this symptom

We compared the Z scores of the basal ganglia and cerebellum between patients showing faciobrachial dystonic seizures versus those without this symptom. The cerebellar vermis showed significantly higher Z scores among patients with faciobrachial dystonic seizures (Mann–Whitney *U* =  − 2.928, *p* < 0.01).

### ^18^F-FDG PET versus brain MRI in pre-treatment phase

Fourteen pre-treatment brain MRIs were available for review. Six patients (42.9%) had abnormal signal intensity on pre-treatment brain MRI suggestive of encephalitis (four patients only in the medial temporal lobes, one patient only in the basal ganglia and one patient in both the medial temporal lobes and basal ganglia). In contrast, all patients in the pre-treatment phase had abnormal FDG uptake on quantified brain analysis using NeuroQ™ in most brain regions.

## Discussion

In this study, we evaluated the pre-treatment and convalescent brain ^18^F-FDG PET images in patients with anti-LGI1 AE. Pre-treatment ^18^F-FDG PETs were abnormal across all patients while brain MRI was abnormal in only 43% of patients. As a group, pre-treatment images using NeuroQ™ showed hypermetabolism in medial temporal cortices, basal ganglia, midbrain, and cerebellum and hypometabolism in areas of the frontal, parietotemporal, and cingulate cortices as well as bilateral Broca regions. In contrast, CortexID™ showed hypometabolism in most brain regions in the pre-treatment phase along with hypermetabolism of the caudate in individual cases. Moreover, comparison of the pre-treatment phase scans with the next available follow-up scan during convalescence using NeuroQ™ showed normalization of these areas of abnormality, except for persistent, though improved, hypometabolism in the left Broca’s region during the convalescent phase. Qualitative assessment of the PET images showed that NeuroQ provides more accurate results. For example, the case provided in Fig. 4a shows mild hypermetabolism in the bilateral medial temporal regions (right greater than left) and NeuroQ^TM^ correctly detects hypermetabolism in these regions (Fig. 4b, slice 46), while CortexID^TM^ shows mild hypometabolism in bilateral temporal associations. The reason of higher NeuroQ^TM^ accuracy compared to qualitative visualization is likely due to smaller brain region analysis, while in CortexID^TM^, larger analyzed ROIs fades the effect of abnormal signal from smaller regions.

Historically, anti-LGI1 AE has been associated with MRI findings of T2 hyperintensities in the medial temporal lobes as well as MRI changes in the basal ganglia in those with faciobrachial dystonic seizures [13]. Prior reports have mentioned abnormal MRI findings in up to 74% of the patients [2]. Brain MRI findings are useful when positive; however, a normal brain MRI would not rule out the possibility of AE [5, 8]. Prior studies have reported MRI sensitivity in the range of 43% [10] to 62% [14] in autoimmune encephalitis, and our result is close to this range. In contrast, out data suggest ^18^F-FDG PET may provide a more sensitive test in the evaluation of patients with suspected AE, particularly among those with anti-LGI1 antibodies.

While prior studies used qualitative methods, several recent studies have evaluated ^18^F-FDG PET in a semi-quantitative fashion based on comparison to a limited number of institutional healthy controls and patients with other diseases. One recent retrospective study of 13 patients (8 with follow-up scans) with comparison to patients with different forms of AE, Alzheimer’s disease, and healthy controls showed that patients with anti-LGI1 were found to have hypermetabolism in the medial temporal lobes, the pallidum, caudate, pons, olfactory, and inferior occipital gyri as well as hypometabolism in several frontoparietal regions [15]. Both medial temporal lobe and putaminal hypermetabolism were reduced after immunotherapy on follow-up convalescent imaging [15]. In a second recent retrospective single-center study, researchers compared ^18^F-FDG PET for 33 patients with varied anti-LGI1 AE clinical presentations to institutional healthy controls and observed a similar pattern of medial temporal lobe, basal ganglia, and cerebellar hypermetabolism with diffuse cortical dysmetabolism [12].

Here, we utilized the commercially available databases NeuroQ™ (which includes fifty health controls) and CortexID™ (which includes over 250 healthy controls) to demonstrate abnormal FDG uptake among all patients during the pre-treatment phase. We observed a pattern of hypermetabolism of the medial temporal lobes and basal ganglia as well as the cerebellum along with hypometabolism in other regions in the pre-treatment phase of anti-LGI1 AE, a more restricted pattern of dysmetabolism that markedly improves during convalescence. These patterns of cortical and subcortical dysmetabolism were more evident using the NeuroQ™ software compared to CortexID™, likely an effect of NeuroQ™ providing a greater number and more restricted regions of analysis than CortexID™. The use of larger regions of interest in Cortex ID™ likely results in the averaging out of areas of abnormal metabolism. For example, the basal ganglia and cerebellum are overall hypermetabolic in our patients when analyzed using NeuroQ™, as has been reported by others, but they appear overall normal using CortexID™.

The diffuse pattern of metabolic changes we observed may in part reflect the diffuse distribution of the LGI1 protein in the human brain, as it has potentially varied roles within a neural network. In a murine transgenic model, LGI1 was expressed by both neuronal and glial cells in the hypothalamus, midbrain, pons, medulla, and cerebellum [16]. Similarly, the 60 kDA isoform of LGI1, which complexes with the KV1.1 potassium channel, was found to be expressed in the temporal neocortex, hippocampus, parietal cortex, frontal cortex, putamen, and occipital cortex [17, 18]. Binding of antibodies to LGI1 in these varied regions may lead to the complex clinical phenotypes observed in patients with anti-LGI1 encephalitis. The refined semiquantitative analysis of FDG-avidity patterns with comparison to a large group of healthy control patients in this study, coupled with the expression of LGI1 in numerous brain regions, potentially expands this association to a network of brain regions.

As an example, several of our patients prior to treatment experienced faciobrachial dystonic movements. The pre-treatment hypermetabolism of the cerebellum in association with faciobrachial dystonic movements that normalizes in convalescence observed here, suggest that these may be acquired dystonias rather than ictal phenomena. Faciobrachial dystonic movements rarely have an ictal correlate on electroencephalopgraphy, and their cessation typically follows the addition of immunotherapy to antiepileptic medications [2, 6]. Furthermore, the cerebellum has been proposed to play a role in network models of dystonia [19], with cerebellar FDG avidity previously observed in patients with dystonia syndromes clinically reminiscent of faciobrachial dystonic movements, including blepharospasm (in association with hypermetabolism of the caudate, cingulate, temporal lobe and hyper/hypometabolism of the thalamus and brainstem structures) and cervical dystonia (in association with hypermetabolism of the putamen, pre-motor cortex, and thalamus) [20].

Clinically, among patients with anti-LGI1 AE, initiation of immunotherapy leads to favorable long-term outcomes [2]. A more recent retrospective study of treatment outcomes among 118 patients with Anti-LGI1 AE demonstrated that corticosteroids are more effective in the acute setting, and immunotherapy provides a more favorable long-term outcome [21]. These studies signify the importance of rapid diagnosis and immunotherapy initiation. The pattern of brain FDG uptake in conjunction with other clinical and laboratory findings may not only provide critical information supporting a diagnosis, but also indicate the likelihood of response to immunotherapy. Follow-up images for six patients in our study demonstrate that areas of dysmetabolism normalized as patients recovered during the convalescent phase (Fig. 5). This suggests ^18^F-FDG PET may also be useful in monitoring recovery from AE. Brain FDG uptake may prove a useful guide to long term management of anti-LGI1 AE, such as informing the decision to continue immunotherapy, and managing symptoms including faciobrachial dystonic movements [5].

The limitations of this study include the following: the study is retrospective with a limited number of patients from one institution. Considering the rarity of this type of encephalitis, large studies are challenging. Another limitation is the inherent low specificity of ^18^F-FDG PET imaging, which is based on the detection of glucose uptake as a measurement of metabolism and is relatively nonspecific; utilizing more specific tracers with the ability to detect inflammation or specific antibodies may provide a better understanding of pathophysiology. Further ^18^F-FDG PET studies with more patients from multiple institutions are warranted to assess the generalizability of our results.

In summary, our findings expand our understanding of the extensive patterns of dysmetabolism in anti-LGI1 AE evident on ^18^F-FDG PET. These are evident in the pre-treatment phase, even in the absence of abnormalities by MRI, and improve in the convalescent phase of anti-LGI1 AE. Thus, ^18^F-FDG PET can potentially be used to guide decisions about early initiation and continuation of immunotherapy for these patients. In addition, these were observed utilizing commercially available software packages, potentially facilitating multi-center studies, our understanding of the underlying neurobiology of AE, and informing patient care. Larger scale, longitudinal ^18^F-FDG PET studies including different subtypes of AE are warranted, which may help differentiate the diverse AE subtypes and suggest treatment response based on the brain FDG uptake pattern.

## References

[1] Irani SR, Michell AW, Lang B, Pettingill P, Waters P, Johnson MR (2011). Faciobrachial dystonic seizures precede Lgi1 antibody limbic encephalitis. Ann Neurol.

[2] van Sonderen A, Thijs RD, Coenders EC, Jiskoot LC, Sanchez E, de Bruijn MAAM (2016). Anti-LGI1 encephalitis: Clinical syndrome and long-term follow-up. Neurology.

[3] Petit-Pedrol M, Sell J, Planagumà J, Mannara F, Radosevic M, Haselmann H (2018). LGI1 antibodies alter Kv1.1 and AMPA receptors changing synaptic excitability, plasticity and memory. Brain.

[4] Dutra LA, Abrantes F, Toso FF, Pedroso JL, Barsottini OGP, Hoftberger R (2018). Autoimmune encephalitis: a review of diagnosis and treatment. Arq Neuropsiquiatr.

[5] Abboud H, Probasco J, Irani SR, Ances B, Benavides DR, Bradshaw M (2021). Autoimmune encephalitis: proposed recommendations for symptomatic and long-term management. J Neurol Neurosurg Psychiatry.

[6] Thompson J, Bi M, Murchison AG, Makuch M, Bien CG, Chu K (2018). The importance of early immunotherapy in patients with faciobrachial dystonic seizures. Brain.

[7] Venkatesan A, Michael BD, Probasco JC, Geocadin RG, Solomon T (2019). Acute encephalitis in immunocompetent adults. Lancet.

[8] Graus F, Titulaer MJ, Balu R, Benseler S, Bien CG, Cellucci T (2016). A clinical approach to diagnosis of autoimmune encephalitis. Lancet Neurol.

[9] Probasco JC, Solnes L, Nalluri A, Cohen J, Jones KM, Zan E (2017). Abnormal brain metabolism on FDG-PET/CT is a common early finding in autoimmune encephalitis. Neurol(R) Neuroimmunol Neuroinflammation.

[10] Solnes LB, Jones KM, Rowe SP, Pattanayak P, Nalluri A, Venkatesan A (2017). Diagnostic value of 18F-FDG PET/CT versus MRI in the setting of antibody-specific autoimmune encephalitis. J Nucl Med.

[11] Probasco JC, Solnes L, Nalluri A, Cohen J, Jones KM, Zan E (2018). Decreased occipital lobe metabolism by FDG-PET/CT: An anti-NMDA receptor encephalitis biomarker. Neurol(R) Neuroimmunol Neuroinflammation.

[12] Li T-R, Zhang Y-D, Wang Q, Shao X-Q, Lv R-J (2021). Recognition of seizure semiology and semiquantitative FDG-PET analysis of anti-LGI1 encephalitis. CNS Neurosci Ther.

[13] Ramanathan S, Al-Diwani A, Waters P, Irani SR (2021). The autoantibody-mediated encephalitides: from clinical observations to molecular pathogenesis. J Neurol.

[14] Baumgartner A, Rauer S, Mader I, Meyer PT (2013). Cerebral FDG-PET and MRI findings in autoimmune limbic encephalitis: correlation with autoantibody types. J Neurol.

[15] Rissanen E, Carter K, Cicero S, Ficke J, Kijewski M, Park M-A et al (2022) Cortical and subcortical dysmetabolism are dynamic markers of clinical disability and course in anti-LGI1 encephalitis. Neurology(R) Neuroimmunol Neuroinflamm 9(2):e1136 10.1212/NXI.0000000000001136 PMC8802686 35091466

[16] Head K, Gong S, Joseph S, Wang C, Burkhardt T, Rossi MR (2007). Defining the expression pattern of the LGI1 gene in BAC transgenic mice. Mamm Genome.

[17] Schulte U, Thumfart J-O, Klöcker N, Sailer CA, Bildl W, Biniossek M (2006). The epilepsy-linked Lgi1 protein assembles into presynaptic Kv1 channels and inhibits inactivation by Kvbeta1. Neuron.

[18] Furlan S, Roncaroli F, Forner F, Vitiello L, Calabria E, Piquer-Sirerol S (2006). The LGI1/epitempin gene encodes two protein isoforms differentially expressed in human brain. J Neurochem.

[19] Prudente CN, Hess EJ, Jinnah HA (2014). Dystonia as a network disorder: what is the role of the cerebellum?. Neuroscience.

[20] Neychev VK, Gross RE, Lehéricy S, Hess EJ, Jinnah HA (2011). The functional neuroanatomy of dystonia. Neurobiol Dis.

[21] Rodriguez A, Klein CJ, Sechi E, Alden E, Basso MR, Pudumjee S (2022). LGI1 antibody encephalitis: acute treatment comparisons and outcome. J Neurol Neurosurg Psychiatry.

